# Feasibility Study of a Powder-Based Supplement Intervention for a future Synbiotic Trial in Breastfed Children from South Africa

**DOI:** 10.21203/rs.3.rs-2842773/v1

**Published:** 2023-05-15

**Authors:** Rupak Shivakoti, Barbara Laughton, Mehr Shafiq, Elisma Schoeman, Richard H Glashoff, Shaun Barnabas, Samantha Fry, Cheng-Shiun Leu, Shuang Wang, Lars Bode, Grace Aldrovandi, Louise Kuhn, Amy L Slogrove

**Affiliations:** Columbia University Mailman School of Public Health; Stellenbosch University Faculty of Medicine and Health Sciences; Columbia University Mailman School of Public Health; Stellenbosch University Faculty of Medicine and Health Sciences; Stellenbosch University; Stellenbosch University Faculty of Medicine and Health Sciences; Stellenbosch University Faculty of Medicine and Health Sciences; Columbia University Mailman School of Public Health; Columbia University Mailman School of Public Health; University of California San Diego; University of California Los Angeles David Geffen School of Medicine; Columbia University; Stellenbosch University Faculty of Medicine and Health Sciences

**Keywords:** Feasibility, synbiotic, infant morbidity, breastmilk, HMO

## Abstract

**Background:**

Children who are HIV-exposed uninfected (HEU), i.e., born to mothers living with HIV despite not acquiring HIV infection themselves, have increased morbidity and mortality. Data suggests that the breastmilk profile, and more specifically human milk oligosaccharide (HMO) composition, differ by maternal HIV status and may partly help explain this increased risk. We are currently conducting an HMO-based synbiotic randomized trial in breastfed children HEU, the MIGH-T MO study (ClinicalTrials.gov Identifier: NCT05282485), to assess the impact on health outcomes of children HEU. Here, we report our experience from a study of the feasibility and acceptability of a powder-based intervention given to breastfeeding children, conducted prior to the initiation of MIGH-T MO.

**Methods:**

10 mothers living with HIV and their breastfeeding children HEU accessing care at Tygerberg Hospital, in Cape Town, South Africa were enrolled. A powder-based product, potato maltodextrin, was mixed with expressed breast milk and administered to the infants daily for 4 weeks. Data on feasibility, acceptability, adherence, and health outcomes were assessed at the enrollment visit and at the 4 week visit, along with weekly telephone calls.

**Results:**

10 mother-infant pairs were enrolled in this study, with infant age ranging from 6–20 months of age. Among the mothers who met the eligibility criteria, all of them enrolled into the study suggesting high acceptability. While there was some Ioss-to-follow-up after the first visit, among the mothers who remained, there were no major feasibility concerns related to study procedures, product administration, adherence, tolerance, and health outcome assessment.

**Conclusion:**

Our pilot study demonstrated that a powder-based intervention for breastfeeding children HEU in South Africa is acceptable and feasible. This suggests potential feasibility and acceptability for other larger studies, including our ongoing MIGH-T MO study, that use similar powder-based interventions such as probiotics, prebiotics, or synbiotics, in breastfed infants from similar settings.

## Background

Infants born to mothers living with HIV have an increased risk of adverse health outcomes despite not acquiring HIV infections themselves. These infants, who are HIV-exposed uninfected (HEU), have higher rates of mortality, respiratory, and gastrointestinal infections, and growth deficits even when the mother is on antiretroviral therapy (ART) (1-9). Whether observed differences in breastmilk composition between mothers with and without HIV can help explain the increased risk of adverse outcomes in infants HEU is an area of active investigation (10-12).

We have observed that maternal HIV status is linked to differences in breastmilk profile, including the composition of human milk oligosaccharides (HMO), and resulting changes in the infant gut microbiome profile (10-12). HMOs are short-chain carbohydrates present in breastmilk and have been linked to improved infant growth and reduced infectious morbidity (13, 14). We hypothesize that these differences in HMO profiles by maternal HIV status partly explain the increased adverse outcomes in infants HEU, and that an intervention of specific HMOs (e.g., 2′-fucosyllactose (2′FL)) given to breastfed infants HEU will reduce these adverse outcomes. To test this hypothesis, we are currently conducting a proof-of-concept, randomized, placebo-controlled trial of a synbiotic intervention (2′FL and a *Bifidobacterium infantis* probiotics) in breastfed infants HEU living in Worcester, South Africa. Details of the ongoing trial, “Mitigating Infectious Morbidity and Growth Deficits in HIV Exposed Uninfected Infants with Human Milk Oligosaccharides” (the MIGH-T MO study, ClinicalTrials.gov Identifier: NCT05282485), can be found in the trial protocol manuscript (15).

While a well-designed randomized controlled trial (RCT) can serve as the gold-standard for determining the causal effect of an intervention, there can be major challenges in the conduct and interpretation of its results in instances of inadequate participant recruitment, follow-up or compliance with study procedures (16). These challenges are especially relevant in studies of probiotic/prebiotic/synbiotic interventions in infant populations as there are potential concerns related to mothers’ acceptance of the intervention and related study procedures. In addition, logistical aspects of successfully administering a powder-based intervention to breastfeeding infants along with details on adherence and morbidity outcome assessments require careful consideration. To address these concerns and avoid potential issues in the MIGH-T MO study to ensure successful recruitment and conduct of the study procedures, we conducted a feasibility study prior to the initiation of the MIGH-T MO study.

In this feasibility study, 10 breastfeeding mothers and children HEU from Cape Town, South Africa were recruited to administer a powder-based product daily for 4 weeks. This product is the placebo as well as the base of the synbiotic intervention in the MIGH-T MO study. The aim of this feasibility study was to determine the acceptability and feasibility of administering a powder-based intervention, and to determine our ability to successfully assess adherence and study outcomes during and at the end of the study.

## Methods

### Feasibility Study Population

In this study, we recruited mother-child pairs accessing care at Tygerberg Hospital, in Cape Town, South Africa. Inclusion criteria were postpartum women living with HIV (WHLIV) of at least 18 years of age, currently breastfeeding and intending to breastfeed for at least the next 6 weeks, with access to a cell telephone for study-related contact, and with children HEU between 1–24 months of age at enrollment. Exclusion criteria were children living with HIV, and severe maternal or child illness (e.g., tuberculosis, major psychiatric or neurological conditions). Study counsellors involved in the community identified potential participants and convenience sampling was used to recruit WLHIV and their children HEU at the study site.

### Feasibility Study Design & Procedures

For this feasibility study, children HEU were administered a powder-based product: potato maltodextrin, which is the placebo for the MIGH-T MO study and the base used to manufacture the synbiotic (i.e., intervention for the MIGH-T MO study). This product was manufactured and packaged into daily-use sachets by International Flavors & Fragrances (IFF). Maltodextrin has been widely used as a placebo in multiple probiotic studies (17, 18), and is also an approved ingredient in infant formula.

**Figure 1:**
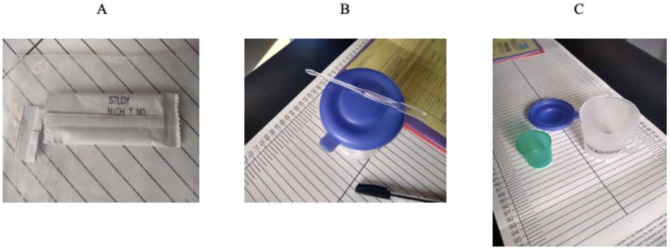
Study Intervention Training Material A: Daily-use sachets of intervention product i.e., maltodextrin. **B:** Stirrer and large medicine cup to collect expressed breastmilk. **C:** Approximately 5mL of expressed breastmilk was transferred from the large medicine cup to the smaller sized medicine cup (green), a stirrer was used to mix in the intervention, and the mixture was cup-fed to the child.

At the enrollment visit at the Family Centre for Research with Ubuntu (FAMCRU), Tygerberg Hospital, the mothers administered the first day of intervention under supervision of the study staff. The staff supported and gave mothers directions on expressing breastmilk, mixing the intervention, and feeding it to their child. During this visit, mothers were directly observed and supported to express breastmilk in a medicine cup which was placed in a larger cup to prevent spillage (Fig. 1C). One sachet of maltodextrin powder (Fig. 1A) was mixed in using a stirrer (Fig. 1B). The mixture was then cup-fed to the child using the medicine cup, with advice on positioning the child’s head to facilitate swallowing.

Maltodextrin powder was packaged in daily-use sachets (1.5 grams per day) and mothers were provided with sachets sufficient for 4 weeks of daily administration, along with the cups and stirrer to take home. A diary and a pen were provided with pre-completed dates to serve as reminders for when to give their child the intervention, when to expect a telephone call from the study staff, and when to come in for their final in-person site visit. Mothers were also encouraged to note down any problems or issues they faced with administering the intervention in space provided in the diary.

After the enrollment visit (week 0), study participants had weekly telephone calls for weeks 1, 2, and 3. During the weekly calls, data on feasibility, acceptability, adherence, and outcome assessment (i.e., child illnesses) were collected. Furthermore, these telephones calls also served to troubleshoot potential issues with the intervention administration or study procedures. Telephone calls were restricted to three attempts to make allowance for mothers not being able to answer their telephones. At week 4, study participants had their last in-person visit at the study site. At this visit, similar data on feasibility, acceptability, adherence, and outcomes were assessed. Mothers were also asked to provide feedback on their overall experience and make suggestions to improve any aspects of the study design.

During all interactions with the mothers, the study staff were advised to take their time talking to the participants and to be cognizant of the mother’s time and comfort in answering questions, especially over telephone calls. To make the mothers familiar with the telephone number and the process of receiving calls from the study team, a telephone call was made in the presence of the mothers while they were at the site. To further facilitate relationship-building between study staff and mothers, follow-up telephone calls were made by the same Research Assistant who initiated the mother’s in-person enrollment visit.

Acceptability was determined by the number of mothers who were screened, met the eligibility criteria, and ended up joining the study without serious concerns around administering the intervention to their children. Feasibility was assessed based on the number of mothers who successfully administered the intervention and whether they faced challenges in administering the product to their children.

To assess adherence, mothers were asked to keep the empty (i.e., used) sachets and bring them to their last study visit. As part of the weekly telephone call, they were also queried on missed doses. In addition, the diary card was reviewed at the week 4 visit, to note date and time of administering the intervention and any concerns they had recorded.

During the study calls and visit, data on child health outcomes, which included healthcare provider visits and any potential illnesses (fever, diarrhea, respiratory illness, or others), along with data on symptoms of intolerance to the placebo (e.g., excessive flatulence, vomiting, irritability, frequency of stool etc.), were collected using a standard questionnaire.

### Feasibility Study Goals and Analysis

The primary goal was to determine the feasibility and acceptability of study product administration. Two indicators were used: 1) among the participants who were eligible, those who agreed to give the intervention to their children, and 2) number of participants who successfully administered the intervention to their children. Further, another goal of this study was to determine the adherence to study intervention, which was analyzed based on the number of doses administered to the child. An additional goal was to determine the feasibility of collecting child health outcome data. This was analyzed based on the ability and response rate to collect illness data in the weekly calls and final study visit.

As this was a feasibility study with mainly qualitative data, we present descriptive and qualitative data. For the study to be considered feasible and acceptable, we expected the majority of mothers to accept this intervention for their child and for the majority of the children to have been administered the intervention successfully.

CONSORT reporting guidelines were used to present this study.

## Results

### Study Population

A total of 10 adult breastfeeding WLHIV and their children HEU were enrolled in this feasibility study from April 2022 to May 2022. Of the 10 children HEU enrolled, 7 were girls and 3 boys, ranging from 6 weeks to 20 months of age.

### Feasibility and Acceptability Recruitment, Enrollment and Acceptability

**Figure 2:**
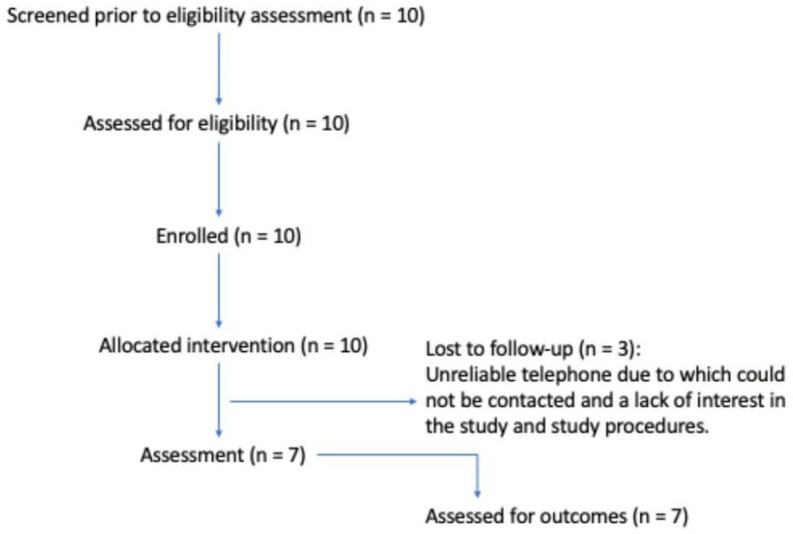
Flow Diagram for the Feasibility Study Flow diagram of progress through phases of the feasibility study–that is, screening, enrolment, intervention allocation, follow-up, and assessment for outcomes.

FAMCRU recruiters identified community members who would meet the inclusion/exclusion criteria, and they were invited to attend screening at our site. All potential participants who were screened met the eligibility criteria. All the mothers who met the eligibility criteria consented and were enrolled, indicating acceptance of the study intervention and procedures for their children (Fig. 2).

### Retention

Seven participants completed most of the study procedures (i.e., attended the weekly telephone calls and the last week site visit). Of these 7 participants, 6 participants completed all telephone calls (and the other participant missed one telephone call), and all 7 completed the final visit. Three participants came in for their first visit only and were thereafter lost to follow-up. One did not have a reliable telephone and could not be contacted after their initial study visit. For the other two participants, a possible reason for loss to follow up was a lack of interest in the study and study procedures.

### Study Product Administration

At their enrollment visits, all 10 mothers indicated acceptance of the intervention and were able to administer the intervention to their child successfully at the study site. Of the 7 mothers who remained on the study, none reported having trouble expressing breastmilk at home. Five of the 7 infants finished most of the milk and powder mix on all days. Of the two infants who did not, one infant did not finish most of the milk and powder mix on 2 days.

The mothers did not experience major challenges in administering it to their children. A few challenges that the mothers experienced included being away from their baby due to work or other reasons which posed a challenge in administering the intervention. Other aspects that were challenging included the children’s resistance to drinking the milk with the powder; one of the mothers reported that her infant did not seem to like the powder and had to be coerced into taking it, and another mother reported that her child was uncooperative in taking the powder when sick or crying.

### Effectiveness of Telephone Calls

Weekly telephone calls to mothers were made to collect data on their infant’s health, challenges or concerns experienced when administering the intervention, and feedback for the study. Two mothers answered their telephone on the first attempt for all calls. Others needed 2 or 3 attempts for contact but did answer their telephones and completed the questionnaire. One mother was reached through a secondary telephone number.

At their last visit, mothers were asked whether the weekly telephone calls were helpful to answer their questions or concerns and 100% of them answered “yes”, citing reasons ranging from having questions about the intervention and their child’s health answered, to the telephone calls serving as reminders to give their child the intervention.

### Outcome Assessments: Infant Health Outcome Assessment

Over the 4 weeks, none of the mothers reported taking their child to visit a doctor or healthcare provider for any symptoms or illness (Table 1). Additionally, none of the children experienced diarrhea, one of the 7 children experienced fever (attributed to teething and not the intervention), and 3 children had respiratory illnesses (cough and cold). One child experienced pimples and constipation for 2 days.

### Adherence Assessment

All 7 mothers who stayed on the study had high adherence in giving their infant the intervention. Mothers were asked if they gave their infant the placebo every day, all mothers except two answered “yes” (Table 1). One of the mothers who said “no”, missed 3 days for reasons likely not related to study products; her child experienced pimples and constipation for 2 days that resolved and did not re-occur after re-initiation of the intervention. Regardless, all mothers on the study gave their infants > 85% of the intervention between the first and last visit.

Intervention adherence was also assessed in terms of the number of opened/used sachets. Mothers were asked to bring in all sachets (both used and unused) at their last study visit (Table 1). Based on the sachets brought in, 5 of 7 mothers gave their infant the powder every day and 2 mothers missed a few doses.

In terms of adhering to intervention guidelines of feeding only one sachet of powder per day, two mothers reported mixing more than one sachet in their expressed breastmilk (Table 1).

### Tolerance Assessment

Overall, the intervention was well-tolerated by the infants with only minor issues reported and common in this age group. Over the 4 weeks, 1 out of 7 infants experienced ‘a lot’ of flatulence prior to one of the telephone calls (Table 1). Two infants experienced ‘a lot’ of spit-up at one timepoint, one of whom concurrently experienced ‘a lot’ of vomit and ‘a bit’ of irritability. Another infant experienced ‘a lot’ of irritability and ‘sometimes’ sleepless nights during one of the study weeks, and only sleepless nights ‘sometimes’ during another study week. None of the infants experienced colic or stomach cramps and none of the infants passed stool more than 3 times in a day in the three days prior to their post-intervention telephone calls/visit.

## Discussion

Our feasibility study of a powder-based intervention administered to breastfeeding infants in South Africa was acceptable and feasible. Breastfeeding mothers were accepting of the intervention for their infants and any concerns that they had were easily addressed by the study staff. There were no major challenges in administering the powder-based intervention to their children, and there was strong participant adherence to study procedures and intervention. Data on infant health status and tolerability were also successfully obtained through site visits and telephone calls. However, there was some loss to follow-up in this study. Overall, our results suggest that a future study of powder-based intervention (e.g., probiotic, prebiotic or synbiotic) for breastfeeding infants in similar settings will likely be acceptable and feasible.

All women screened and eligible to join the study were enrolled, speaking to the high acceptability of the proposed study and intervention. After joining the study, the mothers did not have any prolonged acceptability concerns with regards to the intervention and study procedures. Any initial reservations that they had were either addressed by the study staff or were resolved on their own (e.g., child’s constipation resolved and did not re-occur upon re-starting the study product). The concerns raised were either unrelated to the study product or were due to misunderstandings that were cleared up. For example, some mothers wanted to taste the product themselves first, speaking to the importance of manufacturing extra sachets of products to address such concerns along with training needs.

With regards to the feasibility of administering a powder-based intervention and following the study procedures, all mothers at the initial visit and the rest who were not lost-to-follow-up at the subsequent visits, reported being able to follow them without major challenges or problems. Demonstrating and helping mothers with administering the first dose at the study site was helpful for mothers to troubleshoot and have their questions answered. Our additional training related to breastmilk expression, breast hygiene and preventing contamination underlined the need to educate and support the mothers for successful feasibility.

There was also training for the mothers to maintain breast hygiene including cleaning breasts after expressing milk and cleaning cups properly to prevent contamination. It also helped to keep breast pads in the study room to assist mothers during their in-person visits.

Among the participants that remained in the study, there was high adherence to the intervention. This can be explained partly to the weekly telephone calls that partially served as reminders as well as diary cards that outlined the time and dates for study product intervention for ease of following. Compliance to study procedures and adherence to the intervention is crucial in an RCT and an objective method to quantify this in a powder-based clinical trial is to collect empty sachets. While two mothers on this study discarded their sachets, for future trials it is important to provide consistent reminders to mothers to not discard empty sachets and return them to the study staff.

Collection of outcome data on infant health and tolerability was primarily done through weekly telephone calls. It was feasible to collect this data over telephone calls without difficulty. The ease of telephone calls to collect data makes it a practical mode for ongoing data collection in between study visits to supplement on-site data collection.

Recruitment and retention were dependent on establishing trust between the research institution and participants through building a relationship with the research assistant, providing clear instructions and planning, including considerate call scheduling. During recruitment, study staff were advised to take their time during the consenting and screening phase resulting in all women joining the study among those who were screened and eligible. Diary cards were also useful in structuring the telephone call and follow-up visits. Another reason that likely contributed to study enrollment and retention is financial compensation for the participant’s time on the study. Similar recruitment strategies that were successful will also be used in the MIGH-T MO study.

There was some loss to follow-up in this feasibility study. A likely reason is the relatively short duration of the study and no obvious benefit to the child considering this was a ‘placebo study’. We anticipate higher retention in the main study as there may be prospect for direct benefit to the infants with the synbiotic intervention and additionally, a prolonged connection with the study staff could help with care for the infant.

One challenge related to retention and adherence to intervention experienced in this study, and potentially expected to arise in the main trial, is that of mothers returning to work and leaving their children with caretakers. For this reason, in the main study, it is important to discuss with mothers plans for if and when they will be away from their children on the study and advise them on ways to continue the intervention in her absence e.g., the powder and expressed breastmilk or formula milk can be given to the caretaker to feed to the child.

Despite the valuable information obtained for the future MIGH-T MO trial and similar trials of probiotics in infants, our study has some limitations. One limitation is the limited sample size. While this sample size was informative for the main goals to assess the acceptability and feasibility, the sample size was more limited for the secondary goal of the feasibility of assessing infectious morbidity. Our loss-to-follow-up was also higher than expected but as detailed above some of the issues are likely to be resolved with the design of the main trial. Finally, it remains to be determined whether the feasibility and acceptability will be similar with a bigger sample of mother-infant pairs, and administration of the intervention for a longer time (i.e., 5 months in MIGH-T MO as compared to 1 month for this feasibility study).

This study showed that a powder-based intervention for breastfeeding infants in South Africa was acceptable and feasible. Overall, based on the response to this study, we do not anticipate any other major concerns or challenges with regards to the MIGH-T MO study. We also expect these findings to be useful and likely generalizable to other probiotics/prebiotics/synbiotics studies of breastfeeding infants in similar settings and in other relevant populations (e.g., mothers without HIV from similar settings).

## Conclusion

In conclusion, our study found that administering a powder-based intervention to breastfeeding infants in South Africa was both acceptable and feasible. The intervention was well-received by mothers, who had few concerns that were easily addressed. There were no major challenges in administering the intervention, and participants adhered well to study procedures. While some participants were lost to follow-up, data on infant health and tolerability were successfully obtained through site visits and telephone calls. Overall, our findings suggest that a future study of powder-based interventions, such as probiotics, prebiotics, or synbiotics, for breastfeeding infants in similar settings is likely to be feasible and well-received.

## Figures and Tables

**Table 1:** Outcome Assessments of the Feasibility Study

	Number of Participants
**Infant Health Outcome Assessment**	
Doctor/healthcare visit	0
Diarrhea	0
Fever	1
Respiratory Illness	3
Other*	1
**Infant Tolerance Assessment**	
Flatulence	1
Spit-up	2
Vomit	1
Irritability	2
Sleepless nights	1
Colic or stomach cramps	0
Passed stool more than 3 times in one day	0
**Adherence Assessment**	
Gave placebo every day	5
Followed guideline of feeding only one sachet per day	5
Brought in sachets to the study site	7

## Key messages regarding feasibility

- Uncertainties that existed regarding the feasibility of the MIGH-T MO study were related to potential challenges in participant recruitment, follow-up, and compliance with study procedures, particularly in the context of probiotic/prebiotic/synbiotic interventions in infant populations in South Africa. Logistical aspects of administering a powder-based intervention to breastfeeding infants and assessing morbidity outcomes were also potential concerns. To address these uncertainties and avoid potential issues in the main study, a feasibility study was conducted.
- The key feasibility findings include that the powder-based intervention administered to breastfeeding infants in South Africa was acceptable and feasible. Breastfeeding mothers were accepting of the intervention, and any concerns they had were easily addressed by the study staff. There were no major challenges in administering the powder-based intervention to their children, and there was strong participant adherence to study procedures and intervention. Data on infant health status and tolerability were also successfully obtained through site visits and telephone calls. However, there was some loss to follow-up in this study.
- The feasibility findings suggest that a future study of a powder-based intervention (e.g., probiotic, prebiotic, or synbiotic) for breastfeeding infants in similar settings will likely be acceptable and feasible. The findings can help design the main study with confidence that the powder-based intervention is likely to be acceptable to mothers and feasible to administer to infants.

## Abbreviations

ART: Antiretroviral Therapy
HEU: HIV-Exposed Uninfected
HIV: Human Immunodeficiency Virus
HMO: Human Milk Oligosaccharides
IFF: International Flavors and Fragrances Inc.
FAMCRU: Family Centre for Research with Ubuntu
MIGH-T: MO Mitigating Infectious morbidity and Growth deficits in HIV exposed uninfected infanTs with human Milk
NICHD: Eunice Kennedy Shriver National Institute of Child Health and Human Development
RCT: Randomized Controlled Trial
SAHPRA: South African Health Products Regulatory Authority
WHLIV: Women living with HIV
2’FL: 2’-fucosyllactose
